# 2020 consensus guideline for optimal approach to the diagnosis and treatment of HER2-positive breast cancer in Bosnia and Herzegovina

**DOI:** 10.17305/bjbms.2020.4846

**Published:** 2021-04

**Authors:** Semir Bešlija, Zdenka Gojković, Timur Cerić, Alma Mekić Abazović, Inga Marijanović, Semir Vranić, Jasminka Mustedanagić–Mujanović, Faruk Skenderi, Ivanka Rakita, Aleksandar Guzijan, Dijana Koprić, Alen Humačkić, Danijela Trokić, Jasmina Alidžanović, Alma Efendić, Ibrahim Šišić, Harun Drljević, Vanesa Bešlagić, Božana Babić, Azra Pašić, Anela Ramić, Dijana Mikić, Zlatko Guzin, Dragana Karan, Teo Buhovac, Dragana Miletić, Senad Šečić, Azra Đozić Šahmić, Lejla Mujbegović, Alisa Kubura, Mensura Burina, Nenad Lalović, Nikolina Dukić, Jelena Vladičić Mašić, Mirjana Ćuk, Rajna Stanušić

**Affiliations:** 1 Clinical Center University of Sarajevo, Sarajevo, Bosnia and Herzegovina; 2 University Clinical Center of the Republic of Srpska, Banja Luka, Bosnia and Herzegovina; 3 Cantonal Hospital Zenica, Zenica, Bosnia and Herzegovina; 4 University Clinical Hospital Mostar, Mostar, Bosnia and Herzegovina; 5 University Clinical Center Tuzla, Tuzla, Bosnia and Herzegovina; 6 Cantonal Hospital “Dr. Safet Mujić”, Mostar, Bosnia and Herzegovina; 7 Radiotherapy Center, International Medical Centers, Banja Luka, Bosnia and Herzegovina; 8 University Hospital Foča, Foča, Bosnia and Herzegovina

**Keywords:** HER2, breast cancer, guidelines, treatment

## Abstract

The HERe2Cure project, which involved a group of breast cancer experts, members of multidisciplinary tumor boards (MTB) from health-care institutions in Bosnia and Herzegovina, was initiated with the aim of defining an optimal approach to the diagnosis and treatment of HER2 positive breast cancer. After individual multidisciplinary consensus meetings were held in all oncology centers in Bosnia and Herzegovina, a final consensus meeting was held to reconcile the final conclusions discussed in individual meetings. Guidelines were adopted by consensus, based on the presentations and suggestions of experts, which were first discussed in a panel discussion and then agreed electronically between all the authors mentioned. The conclusions of the panel discussion represent the consensus of experts in the field of breast cancer diagnosis and treatment in Bosnia and Herzegovina. The objectives of the guidelines include the standardization, harmonization, and optimization of the procedures for the diagnosis, treatment, and monitoring of patients with HER2-positive breast cancer, all of which should lead to an improvement in the quality of health care of mentioned patients. The initial treatment plan for patients with HER2-positive breast cancer must be made by a MTB comprised of at least: A medical oncologist, a pathologist, a radiologist, a surgeon, and a radiation oncologist/radiotherapist.

## INTRODUCTION

Breast cancer is the most common female malignancy and one of the leading causes of premature deaths in women worldwide [1]. The exact causes of breast cancer are still unknown, and the identified risk factors that can contribute to the development of this disease are old age, family history, exposure to female reproductive hormones, eating habits, benign breast disease, and environmental factors.

## INCIDENCE OF HER2-POSITIVE BREAST CANCER

**FIGURE 1:**
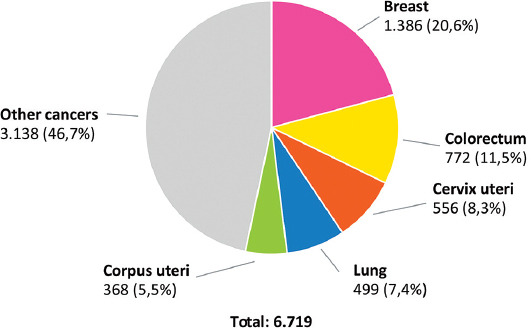
Number of newly diagnosed cancers among women in Bosnia and Herzegovina in 2018 (GLOBOCAN 2018) [1].

It is estimated that there are more than 2 million new breast cancer patients worldwide annually [1]. Every 20 seconds, one woman in the world, is diagnosed with breast cancer [2]. In 2018, as shown in Figure 1, 1386 new cases of breast cancer were diagnosed in Bosnia and Herzegovina, representing 11.6% of all new cases and 20.6% of all cancer cases in women [1].

Human epidermal growth factor receptor 2 (HER2) is overexpressed in 15-20% of all breast cancers. These cancers are so-called HER2-positive breast cancers and are associated with a particularly aggressive course of the disease, high rates of disease recurrence, and increased mortality, if not adequately treated.

## THE APPROACH TO THE DIAGNOSIS AND TREATMENT OF HER2-POSITIVE BREAST CANCER SHOULD BE MULTIDISCIPLINARY

As breast cancer is a very complex disease, and the final outcome depends on the stage of the disease, the biological characteristics of the tumor itself and the initial treatment protocol, patient treatment should be optimally performed at certified Breast Units (specialized departments that treat a large number of patients annually). Recommendations from the EUSOMA (European Society of Breast Cancer Specialists) for the establishment of Breast Units require at least 150 newly discovered breast cancers per year [3]. The organization of breast cancer diagnosis and treatment is based on the teamwork of different specialties gathered in a multidisciplinary tumor board (MTB) whose decisions are based on current professional guidelines. The MTB comprises at least of:

- Medical oncologist
- Surgeon (preferably surgical oncologist)
- Pathologist
- Radiologist
- Radiation oncologist/radiotherapist/oncology and radio-therapy specialist.

If this can be provided at the institution level, it would be optimal for the Board to include an oncology nurse, a physiatrist, and a psychologist/psychiatrist. Given the importance of the surgical oncologist for the final outcome of breast cancer treatment, the panel strongly supports and recommends the introduction of specialization in the field of surgical oncology into the curriculum of specializations for medical doctors in Bosnia and Herzegovina.

The MTB bases diagnosis and treatment decisions on current guidelines and submits them in writing to the patient and her family doctor.

## DIAGNOSIS OF HER2-POSITIVE BREAST CANCER

To make an optimal decision on the treatment and prognosis of HER2-positive breast cancer, adequate and prompt diagnosis is crucial. The optimal pathway for a patient in whom breast cancer has been suspected involves referring the patient to a Breast Unit (if one exists in the competent institution) or to a radiologist specialist.

## RADIOLOGICAL ASSESSMENT AND REPORT

The diagnostic procedure begins at the department of radiology, which is qualified and equipped to accurately detect all breast diseases and is under the competence of a radiology specialist additionally trained to diagnose breast diseases. The diagnostic procedure is based on the so-called “triple procedure,” which includes the following elements:

1. Medical history and clinical examination
2. Diagnostic imaging modalities (ultrasound, mammo-graphy, and magnetic resonance imaging [MRI])
3. Core needle biopsy (CNB).

Medical history is an integral part of the diagnostic procedure and is based on data on previous examinations and other possible breast diseases, menstrual history, gynecological status, administration of hormone replacement therapy, childbirths, family history of breast and ovarian cancer, and information on presenting symptoms.

Diagnostic imaging modalities are based on valid professional guidelines and in accordance with BI-RADS categorization rules. The clinical stage of the disease should be clearly indicated in cTNM format.

The clinical assessment of the breast condition before making a treatment decision should include:

- Clinical examination
- Bilateral mammography (preferably digital native mammography)
- Breast ultrasound
- Core needle or stereotaxic biopsy
- MRI, if available at the institution where the patient is being treated.

Breast ultrasound is of particular importance in patients with a higher amount of fibroglandular stroma (so-called “dense breast”), in women under the age of 40, and in the evaluation of regional lymph nodes, especially the axillary regions.

Mammography is the gold standard in the detection of pathological microcalcifications but becomes less accurate, the amount of dense glandular tissue in the breast increases and should be avoided in younger patients and in some cases in patients with silicone implants.

In accordance with applicable EUSOBI criteria, MRI breast examination is mandatory in the following cases [4]:

1. Pre-operative staging of newly discovered breast cancer
2. Evaluation of the effects of neoadjuvant chemotherapy
3. ccult breast cancer
4. Evaluation of patients with silicone implants
5. Screening of high-risk patients (e.g., lobular cancer and carriers of BRCA 1/2 mutations)
6. Diagnostic dilemmas in case of discrepancies between ultrasound and mammography findings.

CNB is the final step of the triple diagnostic process and is the gold standard in definitive confirmation of the existence of malignant breast disease. The procedure is performed by the radiologist in accordance with applicable professional guidelines. During CNB, 3-6 samples (not less than 3) should be obtained with a 14G (gauge) needle, and sampling time should be clearly noted. The radiologist is obliged to submit, along with biopsy material for histopathological analysis, an accompanying report with information on the localization and size of the biopsied lesion, the possible existence of microcalcifications, the status of regional lymph nodes, and BI-RADS categorization of the biopsied mass. CNBs performed under ultrasound or stereotactic guidance must be performed before initiating any treatment.

A routine evaluation of the estrogen receptor (ER), progesterone receptor (PgR), and HER2 is required for every CNB. The expert panel, while aware of the limitations of interpretation and cutoff values, also recommends routine Ki-67 evaluation for each CNB. The recommended turn-around time is 7 business days.

The clinical assessment of the condition of the axilla before making a therapeutic decision should include:

- Clinical examination
- Ultrasound of axilla
- Ultrasound-guided needle biopsy of suspected lymph nodes
- MRI (depending on the location of the primary tumor).

When tumor spread to regional lymph nodes is suspected, the recommended diagnostic methods include ultrasound examination and MRI examination (depending on the localization of the primary tumor for more precise evaluation of axillary regions and retrosternal lymph nodes along the internal mammary artery). The suspected regional spread of malignancy is confirmed by CNB of the lymph node and histopathological verification.

The assessment of metastatic disease in the initial treatment of early breast cancer is performed on the basis of clinical history and clinical examination. Further tests are not routinely recommended unless there is a high metastatic potential (e.g., locally advanced disease) or the presence of symptoms indicating metastases. The radiologist specialist proves the existence of distant metastases upon the MTB’s decision and according to applicable professional guidelines, with targeted diagnostic tests of the organs, in which metastatic changes occur most frequently (brain, lungs, liver, and bones). These tests include:

- Complete blood count and biochemical blood tests
- In clinically advanced stages (III and IV), and in case of clinical symptoms or abnormal laboratory findings, additional diagnostic procedures (bone scintigraphy and X-ray of pathologically altered and/or painful bones, ultrasound/CT of the abdomen, and X-ray/CT of the lung) may be considered for the purpose of determining the extent of the disease
- Ultrasound/CT of thorax/abdomen
- Bone scintigraphy/X-ray of pathologically altered and/or painful bones
- At the first occurrence of metastases, it is necessary to biopsy them and determine the status of hormonal and HER2 receptors – all metastases, recurrences must be retested if feasible
- PET-CT (acc. to the MTB’s decision and only in the case of non-conclusiveness of other methods).

The above-mentioned radiological examinations must be the sole responsibility of the specialist in diagnostic radiology. The radiologist specialist is obliged to perform all diagnostic procedures (breast ultrasound, mammography, breast MRI, abdominal ultrasound, X-ray, CT, and MRI examinations of all organs suspected of metastatic disease) in accordance with applicable professional guidelines. The MTB will not consider diagnostic findings of primary or metastatic breast tumors of doctors of other specialties valid, and in such cases, the patient will be referred to a competent breast radiologist.

The panel agrees that the evaluation of “tumor markers” (CA 15-3, CA 27.29, CEA) has no validated value in the diagnosis or detection of breast cancer recurrence and recommended that their standard use be excluded from practice at these stages. The evaluation of tumor markers is still recommended in the monitoring of advanced/metastatic breast cancer.

## PRE-OPERATIVE LABELING OF SMALL AND NON-PALPABLE TUMORS

By definition, small tumors are those that measure ≤14 mm in the largest diameter. In the event that the MTB makes a decision about a breast-conserving surgical procedure, the radiologist should mark the exact position of the tumor mass immediately before the surgical procedure in accordance with the applicable professional guidelines and inform the surgeon about this. The standard in marking is the hook wire, and in cases where this cannot be done due to technical reasons, the alternative is to mark the skin while the patient is lying down. The marking of small and non-palpable tumors significantly reduces the need for reoperation due to the positive margins in the operative material.

## TUMOR MARKING BEFORE NEOADJUVANT TREATMENT

The panel emphasizes the great importance of marking the location of the tumor adequately during the CNB (which is the responsibility of the radiologist and/or surgeon). The panel recommends the automatic marker placement during CNB, with an emphasis on multicenter tumors, in which a marker must be placed into each tumor [5]. If the marker cannot be placed during CNB, it can alternatively be placed after confirmation of a malignant tumor, and certainly before the initiation of neoadjuvant systemic therapy. The marker should be placed in the center of the tumor, and any deviation from this requires a detailed description of the location of the marker.

## HISTOPATHOLOGICAL ANALYSIS AND FINDINGS

For a proper histopathological analysis and assessment of HER2 status (and consequently the correct choice of the optimal treatment option for the patient), adequate pre-analytical processing of the sample is crucial, including:

- Minimizing the time to sample fixation (cold ischemia time) to a maximum of 1 h.
- ptimal sample fixation time:
  - 6-72 hours for biopsy samples
  - 12-72 hours for resection samples
- Mandatory use of 10% neutral buffered formalin.

Every pathology laboratory, in collaboration with radiologists and surgeons performing the sampling, should by specific operating procedures define sampling, handling, and transportation of the sample from the sampling site to the pathology laboratory, following the aforementioned pre-analytical phase recommendations and specifics of the central institution organization.

The histopathological report of biopsy material obtained by CNB or surgical resection should be standardized between centers and include the following information (Table 1):

**TABLE 1:**
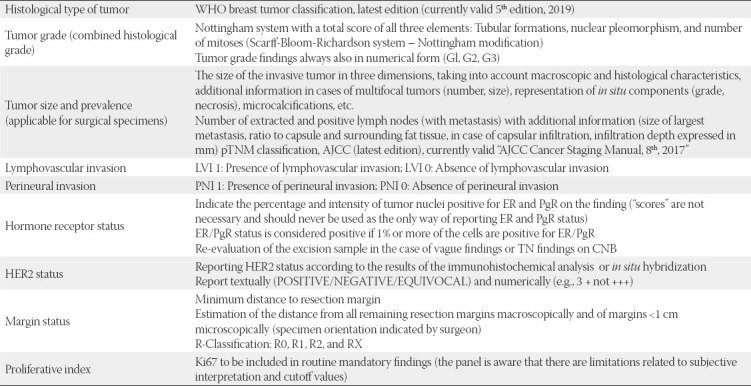
Recommendations for the content of standardized pathohistological findings

The definitive histopathological diagnosis should be made according to the World Health Organization (WHO) classification and the tumor node metastasis (TNM) classification [9].

## DETERMINATION OF HER2 STATUS

The panel unanimously recommends determining HER2 status at the initial diagnosis and first recurrence of each breast cancer, not only for its important prognostic value but also for adequate patient selection for anti-HER2 therapy. HER2 status determination is possible either by measuring the copy number of the HER2 gene using *in situ* hybridization (ISH) or by immunohistochemistry (IHC), which quantifies HER2 receptor on the surface of tumor cells.

Testing is performed according to the latest ASCO/CAP guidelines, the latest version being 2018 [7].

To minimize the number of false positives and false negatives, HER2 testing should be automated and carried out in centers with standardized procedures and control measures for HER2 testing. If testing has been carried out outside such centers, it must be repeated.

Centers that are not automated and/or do not have adequate control measures (external control) should provide adequate conditions within a specified time frame (12 months).

## HER2 ASSESSMENT BY IMMUNOHISTOCHEMISTRY

**FIGURE 2:**
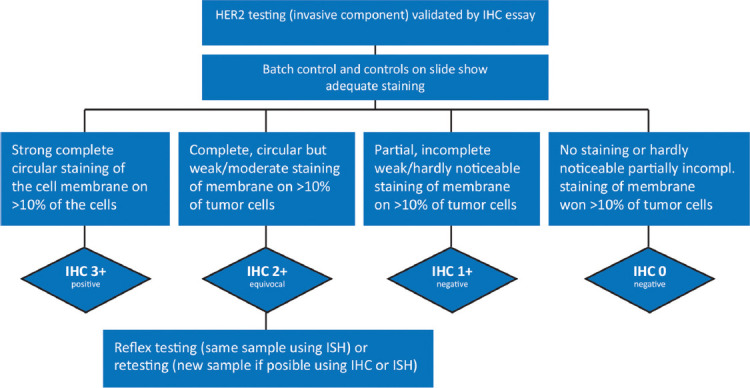
Algorithm for the assessment of human epidermal growth factor (HER2) protein expression by IHC analysis of invasive component of breast cancer sample [10].

The HER2 status determined by the IHC method is expressed by the scores of 3+, 2+, 1+ or 0, or (Figure 2):

In the case of an equivocal result after IHC, the sample must be tested reflexively (same sample by another method) by ISH. Some specific breast cancer subtypes (e.g., micropapillary carcinoma) that immunohistochemically show strong basolateral and lateral partial staining of cell membranes may be considered borderline and need to be tested reflexively by ISH for possibly existing amplification. In some cases of IHC, when ≤10% of tumor cells show strong, circular membrane staining, which is heterogeneous and uneven, discohesive in the tumor, these cases may be considered equivocal HER2 2+ and another sample needs to be provided for (re)testing due to pronounced tumor heterogeneity.

## DETERMINATION OF HER2 STATUS BY IN SITU HYBRIDIZATION (ISH)

HER2 status determined by ISH method is expressed as POSITIVE, NEGATIVE, or EQUIVOCAL, i.e.:

- Positive result**:** HER2:CEP17 ratio ≥2.0 and/or HER2 signals ≥6.
- Equivocal result:
  - IHC: 2+
  - ISH: Signal ≥4-<6
- Negative result: HER2:CEP17 ratio <2.0 and/or HER2 signals <4 [7].

**FIGURE 3:**
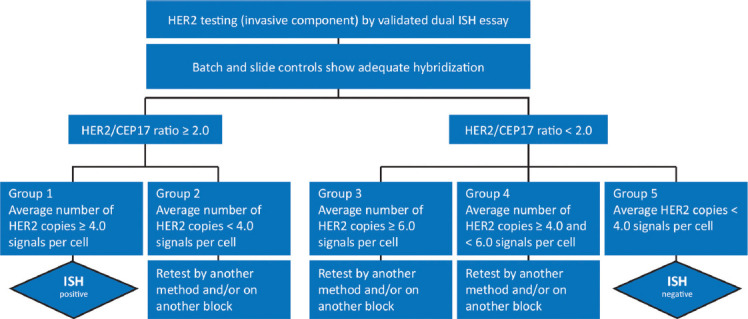
Algorithm for the evaluation of human epidermal growth factor receptor (HER2) gene amplification using the *in situ* hybridization (ISH) method of an invasive component of a breast cancer sample using a double signal test (HER2 gene) [7].

In the case of an equivocal result after ISH, it is necessary to retest the sample by another method and/or on a different block (IHC, if not previously tested) (Figure 3).

If the result is equivocal after IHC and ISH, it should be indicated on the report that both methods are borderline, and the oncologist or MTB decides whether to administer anti-HER2 therapy to the patient [7].

The 2013 ASCO/CAP guidelines, redefined in 2015 and 2018, for determining the HER2 status, recommend that HER2 testing be performed on all CNB samples and repeated on surgical material in the following situations:

- If the HER2 status on needle biopsy could not initially be determined for any reason (insufficient material, technical issues, and problems with pre-analytical procedures, in cases of CNB equivocal results with both methods (ISH and IHH), in cases when surgical resection contains a morphologically distinct high-grade tumor compared to the CNB);
- If the test was positive and in cases of low histologic grade G1 tumors (invasive NOS or low-grade lobular carcinoma, highly hormone-sensitive, invasive tubular, mucinous, cribriform, and adenoid-cystic carcinomas that are by definition negative) [7].

The laboratory performing HER2 testing should continuously monitor the quality of HER2 testing and participate in external, preferably international, and quality assurance testing (e.g., Nordic Immunohistochemical Quality Control (NordiQC) and UK NEQAS). The HER2-positivity rate should be 15% (±5%).

## RADIOLOGICAL – PATHOLOGICAL CORRELATION

After the diagnostic procedures have been performed, the radiologist and pathologist correlate the findings and make judgment about the localization and size of the tumor, the type of tumor, the histological grade, and the biological properties of the tumor. Furthermore, the local and possible regional extent of the disease, as well as the multifocality/multicentricity of the tumor, is evaluated. These data are the basis for proper decisions on the oncological and surgical treatment of malignant breast cancer and are presented by radiology and pathology specialists at the MTB meetings after mutual correlation.

## EARLY HER2-POSITIVE BREAST CANCER THERAPY

The goal of the treatment of early HER2-positive breast cancer is a cure. Patients should receive a complete optimal treatment protocol to maximize the chances of being fully cured. The choice of treatment strategy is the decision of the MTB, taking into account the wishes of the patient, whose therapeutic options should be explained clearly and in detail. The optimal treatment approach must be based on important prognostic and predictive factors, such as tumor size, number of lymph nodes involved, tumor grade and histological type, HER2 and hormonal status (ER/PgR), Ki-67, comorbidities, lymphovascular and perineural invasion, as well as age and general status of the patient.

Before initiating HER2-positive breast cancer treatment, the following basic work-up should be performed for each patient:

- CBC, blood glucose
- Kidney and liver function tests
- ALP
- Ca
- Heart ultrasound (left ventricular ejection fraction [LVEF]).

## NEOADJUVANT SYSTEMIC THERAPY FOR PATIENTS WITH EARLY AND LOCALLY ADVANCED HER2-POSITIVE BREAST CANCER

Treatment of early breast cancer may begin either by surgery followed by adjuvant systemic treatment or by neoadjuvant (pre-operative) systemic therapy. Neoadjuvant systemic therapy can help convert tumor from inoperable to operable stage, increase the chance of breast-conserving surgery (BCS) and less radical axillary dissection, reduce the morbidity associated with breast surgery, and assist, along with other risk factors, in assessing the prognosis of the disease. In general, studies have shown that there is no difference in overall survival if the same systemic treatment protocol (same regimen, same number of cycles) is administered neoadjuvantly or adjuvantly, but achieving pathologic complete response (pCR) after administration of neoadjuvant systemic therapy is associated with an improvement in survival [8]. HER2-positive status, along with other factors such as negative hormonal status, G3, N0, cT1/cT2, is a strong predictive factor for the response to neoadjuvant therapy (NAT), and therefore anti-HER2 therapy should preferably be initiated already at the neoadjuvant stage, to increase the chance of achieving pCR.

All patients with HER2-positive early and locally advanced breast cancer that are candidates for adjuvant systemic therapy are also candidates for neoadjuvant systemic anti-HER2 therapy [8] regardless of tumor size, especially in the following cases:

- N-positive tumor
- Patient’s desire for BCS, which is not possible without reducing the tumor size with systemic therapy first
- Inoperable tumor
- Inflammatory breast cancer [9].

Neoadjuvant anti-HER2 therapy today is the standard in the treatment of HER2-positive early and locally advanced breast cancer. Anti-HER2 therapy should be started as soon as possible, preferably without anthracyclines, so as not to delay the administration of targeted anti-HER2 therapy. Anti-HER2 therapy in neoadjuvant treatment should always be administered in combination with chemotherapy and never as monotherapy or as a combination of two anti-HER2 agents.

The preferred protocol in the neoadjuvant treatment of HER2-positive breast cancer is the combination of pertuzumab + trastuzumab with chemotherapy (Table 2), based on the results of the NeoSphere and TRYPHAENA clinical trials, which showed that the administration of dual blockade of trastuzumab + pertuzumab with chemotherapy in neoadjuvant setting significantly increased the rate of pathologic complete response (pCR) compared to the administration of a single anti-HER2 drug with chemotherapy. In the NeoSphere clinical trial, the dual blockade trastuzumab + pertuzumab in combination with docetaxel resulted in the pathological complete response rate of tumors in the breast and in the axillary and regional lymphatics (pCR) of 45.8% compared to 29.0% and 26.0% when docetaxel was combined with only trastuzumab or pertuzumab, respectively [10,11].

**TABLE 2:**
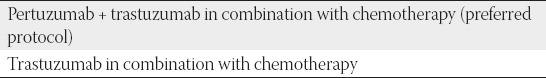
Recommended therapy protocol in neoadjuvant HER2-positive breast cancer treatment

In the TRYPHAENA clinical trial, the effect of dual HER2 blockade trastuzumab + pertuzumab in combination with different chemotherapy regimens was examined. In the first group of patients, dual blockade was combined with the docetaxel/carboplatin combination, the second group of patients received trastuzumab + pertuzumab in combination with docetaxel after four cycles of anthracycline-based protocol, while in the third group, the dual blockade was administered concomitantly with anthracycline protocol and docetaxel. In all three groups, the pCR rate was over 54%, with the highest pCR rate of 63.6% being in the group of patients treated with dual blockade in combination with docetaxel and carboplatin [12]. In patients with hormone receptor-negative (HR-) breast cancer, pCR rates were as high as 83.8% depending on the protocol they received, and it is expected that these patients would benefit most from the use of dual blockade pertuzumab + trastuzumab in clinical practice [12,13].

Based on the results of the NeoSphere and TRYPHAENA clinical trials, panel members concluded that the administration of dual HER2 receptor blockade trastuzumab + pertuzumab with chemotherapy in NAT is the optimal treatment option, especially in patients at high risk of disease recurrence.

Similar recommendations on the application of dual blockade in neoadjuvant setting are also made by ESMO, NCCN, and St. Gallen guidelines [5,6].

Given that comparative trials have absolutely demonstrated that the intravenous and subcutaneous formulations of trastuzumab have comparable efficacy and pharmacokinetic profile as well as similar safety profiles [13], the panel suggests that any patient who is a candidate for trastuzumab therapy can receive either of the two formulations, in accordance with the judgment of the treating physician and the personal preferences of the patient.

## ASSESSMENT OF THE RESPONSE TO SYSTEMIC NEOADJUVANT THERAPY

The response to systemic NAT may be evaluated during and after the administration of systemic NAT. Manual palpation and radiological modalities (mammography and MRI) are basic methods in monitoring the efficacy of systemic NAT and the identification of potential disease progression. All planned systemic NAT should be completed before surgery unless disease progression occurs during treatment. The final evaluation of the efficacy of NAT is performed histopathologically, on a surgical specimen, if the tumor is operable after NAT.

## RADIOLOGICAL ASSESSMENT OF THE RESPONSE TO SYSTEMIC NAT

The most precise diagnostic modality for monitoring the response to NAT is an MRI breast examination, which should be performed before, halfway through, and after neoadjuvant treatment. The radiologist estimates the size of the residual tumor based on RECIST criteria and reports on the morphology and intensity of the radiological response.

According to morphology, response to therapy can be concentric and defragmented, and in intensity, it can be complete or partial. A partial response to therapy may be good (in case the tumor is smaller by 50% or more than the initial measurement) and poor (in case the tumor diameter is larger than 50% of the initial tumor size). The next category is “stable disease,” i.e., there is no therapeutic response and the last option is disease progression (when the tumor diameter is larger by 20% or more than the initial measurement before NAT). It is important to note that MRI screening is a radiological method of assessing therapeutic response that can both underestimate and overestimate the true size of the residual tumor during NAT. Precise assessment is possible after radiological-pathological correlation of neoadjuvant treatment response.

If MRI is not available at the center where the patient is being treated, the response to systemic NAT may be assessed by manual palpation, breast/axillary ultrasound and/or bilateral mammography. One patient should be monitored by the same radiological method. It is not recommended to use PET-CT in the assessment of the effect of NAT.

## HISTOPATHOLOGICAL EVALUATION OF THE RESPONSE TO SYSTEMIC NAT

Histopathological evaluation of breast tissue and residual disease after systemic NAT is a key component of evaluating the effect of systemic NAT and should be performed in close correlation and comparison with clinical and radiological findings. Clinical facilities should have clearly defined procedures for the labeling and orientation of all segmentectomy/mastectomy surgical specimens sent for PH analysis. Histopathological report after NAT should include the following:

- The yp prefix with the TNM classification.
- Identification of tumor bed macroscopically and microscopically in correlation with radiological findings.
- Report on ypTN after NAT – determination of the largest macroscopically and microscopically measured tumor focus.
- Reporting of tumor size as a total extent of tumor bed area involved by infiltrates of residual vital invasive carcinoma.
  - Given that residual disease has prognostic value, the determination of the residual cancer burden (RCB) score is of importance in characterizing the effect of treatment when pCR has not been achieved. MD Anderson RCB score is calculated on the basis of the fraction of tumor bed involved by invasive carcinoma without *in situ* component, the dimensions of tumor bed involved by residual cancer, the number of residual disease-positive lymph nodes, and the largest diameter of the largest residual nodal metastasis. The RCB score is expressed on a scale of 0-3, where 0 represents the pCR and the scores 1-3 represent the progressively greater residual disease. The RCB score has a proven validity independent of the yAJCC stage after NAT.
- A clearly indicated therapeutic effect:
  - Lack of therapeutic effect or minimal response,
  - Partial tumor regression, or
  - Complete regression-pCR if there is no residual invasive carcinoma, angioinvasion, and lymph node metastasis.

After the completion of NAT, it is not necessary to retest ER/PgR and HER2 status by IHC.

If systemic NAT with anti-HER2 therapy has not resulted in satisfactory response and cancer remains inoperable, it is recommended that treatment be continued with neoadjuvant radiotherapy concomitantly with trastuzumab.

## SURGICAL TREATMENT OF EARLY HER2-POSITIVE BREAST CANCER

Surgery is one of the keys and essential steps in the treatment of early HER2-positive breast cancer, and primary (neoadjuvant) systemic therapy should always be followed by surgery. Surgical treatment aims for adequate resection with clean resection margins and includes:

- Breast-conserving procedures, and
- Radical procedures do not preserve breast tissue (mastectomy with or without primary reconstruction).

If BCS is planned, it is crucial to mark the primary tumor with a marker to allow adequate surgical access.

## CHOICE OF SURGICAL PROCEDURE AFTER SYSTEMIC NAT

### Breast surgery

In general, the survival rate after BCS followed by radiation therapy is equivalent to the survival rate after (modified) radical mastectomy. BCS should be the surgical treatment of choice after systemic NAT, but only if microscopically clean margins can be ensured. The optimal period for performing surgery after NAT completion in HER2-positive breast cancer is within 3 weeks.

Mastectomy after NAT is indicated in cases of:

- Positive tumor margins after repeated excision,
- When radiotherapy cannot be performed,
- Inflammatory breast cancer,
- The explicit wish of the patient,
- A large tumor relative to breast size,
- Multicentric tumors,
- If it is not possible to identify the primary tumor lesion after CR due to lack of placement of the clip [6].

It is important to mark the resected breast tissue as well as any subsequently resected tissue for the orientation of the pathologist in regards to resection margins. The assessment of the extent of disease in axillary lymph nodes is mandatory [5].

## AXILLARY SURGERY

In clinically positive enlarged axillary lymph nodes or a suspicious lymph node visualized by ultrasound, it is necessary to perform cytological aspiration or biopsy of the node.

In clinically negative stage I, IIA, IIB, and IIIA breast cancer axilla, it is recommended to perform a sentinel lymph node biopsy (SLNB), which represents the gold standard in axillary staging in early breast cancer with clinically negative lymph nodes [6]. Randomized clinical trials have shown that this approach, compared to standard axillary dissection, leads to a reduction in arm and shoulder morbidity (pain, lymphedema, and loss of sense of touch).

SLNB is preferably performed after systemic NAT.

The current gold standard in implementing SLNB is the use of dual marking: Tc-99m radioisotopes and blue color. In case of histopathological verification of macrometastases in one or two sentinel lymph nodes, when BCS and post-operative radiotherapy are planned, as well as in cases where sentinel lymph nodes are negative, axillary dissection is not necessary [14]. In cases of metastasis verification in the sentinel lymph node in patients that are scheduled for a mastectomy, axillary dissection should be performed.

Level I/II axillary dissection according to Berg (minimum 10 nodes) should be performed in case of:

- Confirmation of positive lymph nodes using FNA, CNB, or SLNB;
- After a failed attempt to identify the sentinel lymph node; and
- After neoadjuvant chemotherapy has been administered, dissection is not mandatory; in case of negative axilla, sentinel biopsy procedure may be performed after NAT, but the identification of 3-4 sentinel lymph nodes is desirable.

**FIGURE 4:**
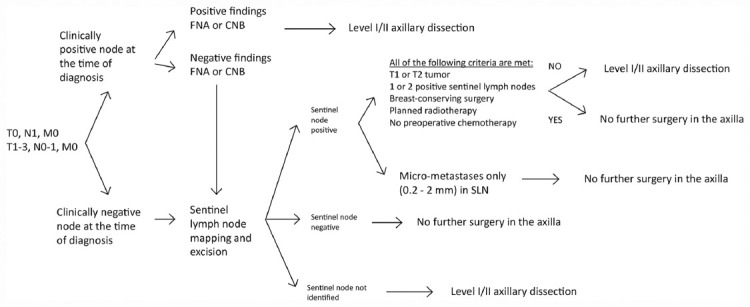
Surgical staging and axillary dissection algorithm.

If the infiltration of lymph nodes in the third level of the axilla is verified intraoperatively, dissection of the third level of the axilla is indicated where possible (Figure 4).

## ADJUVANT HER2-POSITIVE BREAST CANCER THERAPY

The role of adjuvant (post-operative) therapy is to eliminate micrometastases that persist after surgery and to prevent disease recurrence. Early-stage therapy for HER2-positive breast cancer aims to cure. If therapy is unsuccessful, distant metastases can occur and breast cancer becomes incurable.

Given the aggressive nature of HER2-positive breast cancer, anti-HER therapy (trastuzumab/trastuzumab + pertuzumab) should be initiated as soon as possible (within 3-6 weeks) after surgery. The administration of anti-HER therapy should be initiated concomitantly with taxane-based therapy. Clinical studies have shown that the optimal duration of adjuvant anti-HER2 therapy is 1 year (or up to a total duration of treatment with targeted anti-HER2 therapy of 1 year if the patient receives anti-HER2 therapy in the neoadjuvant setting).

## SYSTEMIC ADJUVANT ANTI-HER2 THERAPY PROTOCOLS RECOMMENDED

If the patient received 4-8 cycles of anthracyclines and taxanes in the neoadjuvant stage, even in the absence of pCR, no further chemotherapy is required in adjuvant treatment, and only anti-HER2 therapy should be continued up to a total of 1 year. Preferred adjuvant-stage anti-HER2 therapy protocols are shown in Table 3:

**TABLE 3:**
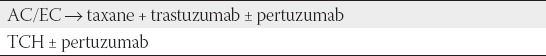
Preferred protocols in the adjuvant treatment of HER2-positive breast cancer

Adjuvant administration of trastuzumab is recommended in all patients with early HER2-positive breast tumors >1 cm. In tumors of 0.6 cm-1 cm with negative lymph nodes, as well as HER2-positive tumors smaller than 0.6 cm with micrometastases in axillary lymph nodes, trastuzumab should also be administered adjuvantly.

Although the introduction of trastuzumab for 1 year in combination with chemotherapy in (neo) adjuvant treatment of early HER2-positive breast cancer has led to tremendous improvement, 1 in 4 patients will still experience disease recurrence within 10-11 years of diagnosis [14], and more than 50% of those who receive a combination of pertuzumab and trastuzumab with a taxane in the first-line treatment of HER2-positive metastatic breast cancer will die within 5 years. Patients with lymph node-positive and hormone receptor-negative disease have a particularly high risk of disease recurrence [15].

Considering this, along with the results of the APHINITY trial, adjuvant treatment of patients with HER2-positive breast cancer at high risk of disease recurrence (especially N+ and/or HR-disease) should include pertuzumab in addition to trastuzumab. The APHINITY trial included 4804 patients and found that adding pertuzumab to a combination of trastuzumab and chemotherapy reduced the risk of invasive breast cancer recurrence by 23% in patients with N+ disease and 24% with HR- disease. In adjuvant treatment, pertuzumab should be administered in combination with trastuzumab for 1 year (up to a total of 18 cycles, disease recurrence or unacceptable toxicity) as part of comprehensive treatment of early breast cancer, regardless of the time of surgery [15].

## PATIENTS WITH RESIDUAL DISEASE AFTER NEOADJUVANT ANTI-HER2 THERAPY

In a subset of patients, systemic neoadjuvant treatment of early HER2-positive breast cancer does not result in pCR and there is residual disease present in the breast and/or axilla. CTNeoBC meta-analysis showed that pCR is associated with an improvement in long-term clinical outcome and those patients with residual disease after NAT have a higher risk of disease recurrence [16]. In such patients with residual cancer after systemic NAT, therapy should be escalated and 14 cycles of trastuzumab emtansine (T-DM1) should be administered.

The recommendation for this approach is based on the results of the KATHERINE trial, which showed that adjuvant treatment with T-DM1 in patients who had residual disease after neoadjuvant chemotherapy and HER2-targeted therapy reduced the risk of recurrence of invasive breast cancer or death by 50% compared with those who continued treatment with trastuzumab alone [17].

The estimated percentage of patients without invasive disease at 3 years was 88.3% in the T-DM1 versus 77.0% in the group that received trastuzumab alone [17]. A clear and consistent treatment benefit was observed in all key clinically relevant subgroups, regardless of the size of the residual tumor, hormone receptor (HR) status, lymph node status, and previous use of dual HER2 blockade (pertuzumab-trastuzumab) [17].

If treatment with T-DM1 is discontinued due to toxicity, patients are advised to continue the administration of trastuzumab (with or without pertuzumab) to complete a total treatment period of 1 year [6]. Anti-HER2 therapy may be administered concurrently with radiotherapy and endocrine therapy if indicated.

**FIGURE 5:**
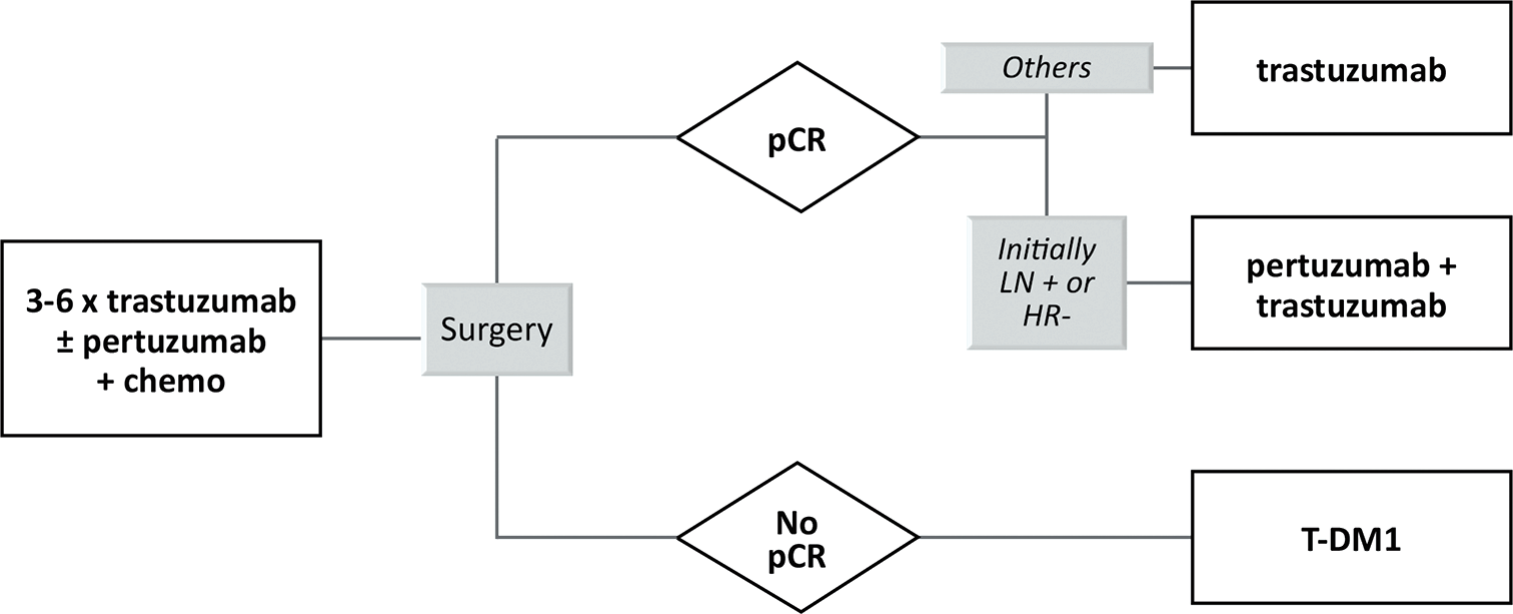
Algorithm of adjuvant anti-HER2 therapy after neoadjuvant treatment, taking into consideration pathologic complete response as a decision point for further therapy [17].

Optimal adjuvant anti-HER2 treatment approach based on response to systemic NAT is shown in Figure 5.

## MONITORING CARDIAC FUNCTION DURING ADMINISTRATION OF ANTI-HER2 THERAPY

In the administration of drugs that inhibit HER2 receptor activity, a well-described side effect is a cardiotoxicity, which may manifest either as an asymptomatic decrease in LVEF or as a symptomatic failure of cardiac function. For this reason, evaluation of cardiac function, i.e., measurement of LVEF before the initiation of anti-HER2 therapy and every 3 months during anti-HER2 therapy, is recommended [15].

Treatment with trastuzumab-pertuzumab may be initiated if the initial LVEF is >50%. If LVEF drops by 10 or more points below baseline or to <50% during treatment with trastuzumab-pertuzumab, therapy should be temporarily withheld. LVEF evaluation should be repeated within 3 weeks and, if the LVEF value recovers to >50% or to <10 units compared to pre-treatment value, trastuzumab-pertuzumab therapy may be resumed. If LVEF does not improve or decreases even further, and if symptomatic congestive heart failure develops, permanent discontinuation of anti-HER2 therapy should be considered. All such patients should be referred for cardiac specialist [15].

It should be noted that cardiotoxicity associated with the administration of anti-HER2 therapy is reversible in nature and that standard ACE inhibitor therapy in most cases leads to an improvement in cardiac function and recovery of LVEF, after which most patients can resume planned anti-HER2 therapy.

The recommendations for the management of patients receiving trastuzumab-pertuzumab based on LVEF measurements are shown in Table 4.

**TABLE 4:**
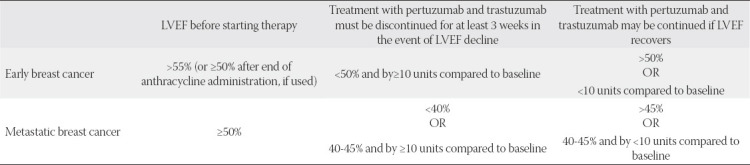
Trastuzumab/pertuzumab dual therapy initiation/discontinuation criteria

## ADJUVANT BREAST CANCER RADIATION THERAPY

The goal of adjuvant (post-operative) radiotherapy for HER2-positive breast cancer is to improve local disease control and reduce relapse rates. If radiotherapy is performed after systemic NAT, the decision on radiotherapy treatment should be based on the pre-treatment clinical stage, and the following parameters listed in the histopathological report and tumor characteristics should be considered (Table 5):

- Presence of residual tumor after NAT,
- Number of lymph nodes involved,
- Relation of lymph node metastasis to node capsule,
- Infiltration of perinodal tissue and
- The presence of any of the high risk factors for local recurrence.
- Risk factors for recurrence and disease dissemination include:
- Central tumor localization,
- High histological grade (G3),
- Negative ER status,
- (Focally) positive resection margins (for a greater positivity of resection margins, reoperation should be recommended),
- The presence of lymphovascular and/or perineural invasion and
- Younger age (<50 years).

**TABLE 5:**
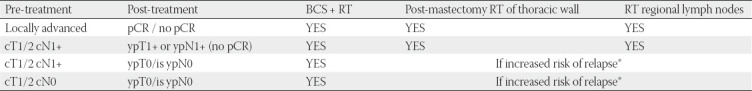
Radiation therapy based on pre- and post-systemic NAT status

Whole breast irradiation is recommended in all cases where a BCS is performed. The omission of breast radiotherapy after BCS for HER2-positive breast cancer is not recommended in any case, even when other conditions for the omission of irradiation are met (expected survival <10 years, pT1, pN0, R0, HR+) (Table 6).

**TABLE 6:**
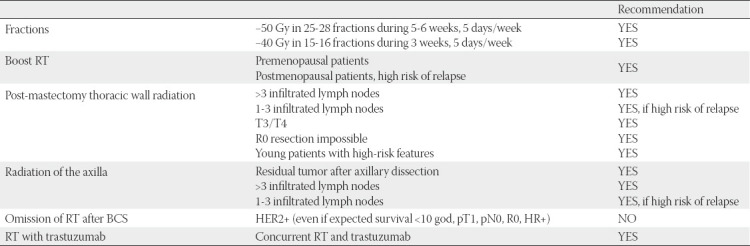
Radiation therapy based on pre- and post-systemic NAT status

The irradiation field includes the remaining part of the breast. The recommended total dose is 45-50.4 Gy in 25-28 fractions or 40-42.5 Gy in 15-16 fractions (hypofractionated radiation). The radiation is performed in daily doses of 1.8-2 Gy, 5 days a week for 5-6 weeks, or 2.5-2.67 Gy during 5 days a week for 3 weeks in the hypofractionated protocol.

In cases of high risk of disease recurrence after BCS (regardless of postmenopausal status), a radiation boost of the tumor bed at a dose of 10-16 Gy in 4-8 fractions is recommended [18].

Lymph node radiotherapy is recommended, in case 4 or more lymph nodes are involved. There is still a dilemma for 1-3 positive lymph nodes, but radiotherapy may be considered in this case as well. In such cases, all the above-mentioned high-risk parameters should be considered, and the presence of extranodal extension of the disease should be taken into consideration. There are also high-risk cases with N0 status, in which the irradiation of lymphatic drainage regions (only axillary, without supraclavicular area) should be considered. In case of micrometastases in SLNB, axillary irradiation without dissection is recommended. For micrometastases, it is not recommended to dissect and irradiate the axillary region.

Thoracic wall radiation after a mastectomy is recommended in cases of locally/locoregionally advanced disease (T3/T4 primary tumor with >3 involved lymph nodes or 1-3 lymph nodes in the presence of other risk factors), if R0 resection is not possible and if the patient is of younger age.

For an occult breast tumor diagnosed on the basis of the presence of metastases in the axillary lymph node, irradiation of the entire breast and axilla is recommended unless a mastectomy has been performed (then only irradiation of the lymphatic drainage region, according to indications, is recommended).

Radiotherapy is safely administered concurrently with trastuzumab (and hormone therapy) and they are considered to have a synergistic effect. Sophisticated irradiation techniques, such as breathing-synchronized radiation, allow for a significant reduction in incidental dose delivery to the heart in the left-sided tumor localization. This is extremely important for patients receiving cardiotoxic therapies such as trastuzumab or anthracyclines [18].

## PATIENT FOLLOW-UP AFTER ADJUVANT TREATMENT OF EARLY HER2-POSITIVE BREAST CANCER

Following adjuvant treatment, it is necessary to follow-up patients, with the main purpose of detecting early local recurrences or contralateral tumor and to monitor treatment side effects (Table 7).

**TABLE 7:**
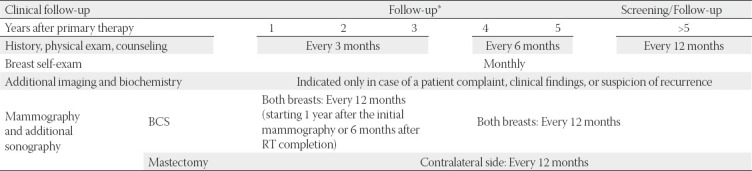
Patient follow-up after adjuvant treatment

The proactive search for distant metastases using radiological methods (bone scintigraphy, liver ultrasound, X-ray, CT, and PET scans) should not be done, due to unnecessary patient irradiation as well as the unproven clinical benefit of early detection of asymptomatic metastases in terms of overall survival. Furthermore, the use of tumor markers in monitoring and detecting HER2-positive breast cancer recurrence has no proven value and should not be used for this purpose.

In addition, patients should be motivated to adhere to adjuvant endocrine therapy (if indicated), provided psychological support for the return to a normal life after breast cancer, and be counseled and supported in leading a healthy lifestyle, including dietary modification and physical activity [6].

## LOCOREGIONAL DISEASE RECURRENCE

In case of locoregional disease recurrence after adjuvant therapy, the following test (as with initial primary tumor treatment) should be done before any further treatment decision:

- Biopsy,
- Hormone receptor status
- HER2 status
- Complete re-staging.

Following diagnostic tests, further treatment options include surgery and radiation therapy as well as systemic therapy, based on the initial treatment and the decision of the MTB (Table 8) [6,7].

**TABLE 8:**
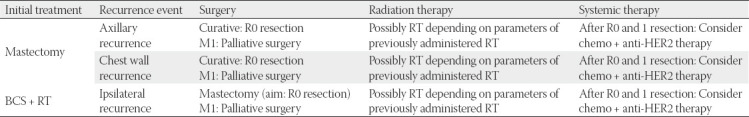
Treatment options for locoregional recurrence of HER2+ breast cancer

## TREATMENT OF METASTATIC HER2-POSITIVE BREAST CANCER

The goal of treating metastatic HER2-positive breast cancer is to extend the time to disease progression and overall survival, and to maintain quality of life, symptom control, and prevent serious complications [6,7]. Systemic therapy of metastatic HER2-positive breast cancer (anti-HER2 therapy with chemotherapy) is the treatment of choice over local surgery and radiotherapy, which have a proven benefit in the palliation of symptoms in disseminated disease. A distinct entity is an oligometastatic disease, in which local metastasis treatment is of greater importance and may delay the initiation of systemic therapy.

## LOCAL METASTATIC HER2-POSITIVE BREAST CANCER THERAPY

### Surgery

The decision on local surgical treatment for metastatic HER2-positive breast cancer should be made by the MTB, taking into account the patient’s wishes. Surgical removal of the breast in HER2-positive metastatic breast cancer does not lead to an improvement in survival (except in patients with bone metastases only).

Breast surgery in metastatic HER2-positive disease is generally not recommended, and “palliative” mastectomy may be performed in selected patients (younger age, inability to tolerate systemic therapy) to improve quality of life or if this is patient’s explicit wish. It is also indicated in patients with the oligometastatic disease who had a good response to systemic therapy [6,7].

In the presence of solitary or small numbers of CNS metastases, metastasectomy (with or without whole-brain radiation) may be considered if R0 resection is possible.

Resection of liver metastases is generally not recommended since studies have not demonstrated the benefit of local treatment versus systemic chemotherapy [6,7,19,20] and could only be considered in HER2-positive patients under the age of 50 with solitary metastasis <5 cm.

### Radiotherapy

Local radiotherapy for metastatic HER2-positive breast cancer may be administered after surgery in *de novo* metastatic disease or as the only local therapy, without prior surgery. The role of radiotherapy in the polymetastatic stage of HER2-positive breast cancer is the palliation of pain and the prevention of complications (e.g., pathologic fractures as a consequence of bone metastases). In selected cases of patients with HER2-positive oligometastatic breast cancer, the role of stereotactic radiotherapy may potentially be curative.

Depending on the localization and prevalence of metastases in the CNS, the following irradiation options shown in Table 9 are possible.

**TABLE 9:**
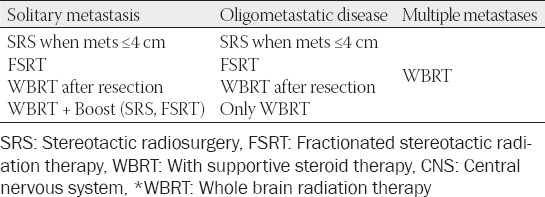
Radiation treatment options based on localization and the extent of CNS metastases

Stereotactic radiotherapy in oligometastatic disease is also performed outside the CNS (when it is called SBRT, stereotactic body radiotherapy), most commonly in the lungs, liver, bones, and lymph nodes. For each region, there are specific dose and fractionation method recommendations.

## SYSTEMIC METASTATIC HER2-POSITIVE BREAST CANCER THERAPY

Systemic anti-HER2 therapy with chemotherapy is the therapy of choice for metastatic HER2-positive breast cancer. Before the initiation of systemic therapy, the observed metastatic changes should be (re)biopsied to confirm the diagnosis as well as the status of hormonal and HER2 receptors. Anti-HER2 therapy should be initiated promptly, in the first line of HER2-positive metastatic breast cancer treatment. In case of disease progression during anti-HER2 therapy, the patient should continue anti-HER2 therapy, optimally with the replacement of the anti-HER2 agent. Treatment with anti-HER2 therapy in the metastatic stage should be administered until disease progression or unacceptable toxicity.

## FIRST-LINE THERAPY FOR METASTATIC HER2-POSITIVE BREAST CANCER

The gold standard in the first-line treatment of metastatic HER2-positive breast cancer is dual blockade (trastuzumab-pertuzumab) in combination with chemotherapy (Table 10). This recommendation is supported by all global treatment guidelines (ESMO, AGO, NCCN, St. Gallen) as well as the expert panel and is based on the results of the CLEOPATRA clinical trial. This study, which included 808 patients in first-line treatment of metastatic HER2-positive breast cancer, showed that the addition of pertuzumab led to a statistically significant improvement in progression-free survival (PFS) compared to trastuzumab and chemotherapy alone (from 12.4 to 18.5 months) and to an improvement in median overall survival (OS) from 40.8 to 56.5 months. The risk of death was reduced by 34% with the addition of pertuzumab to the combination of trastuzumab and chemotherapy [21,22].

**TABLE 10:**
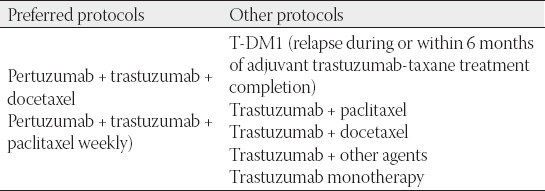
Preferred protocols in first-line treatment of metastatic HER2+ breast cancer

Trastuzumab alone in combination with various chemotherapy agents or as monotherapy is also a valid option in the first-line treatment of metastatic HER2-positive breast cancer. A special group of patients comprises patients who have relapsed during or within 6 months of adjuvant anti-HER2 treatment, in whom T-DM1 is the therapy of choice in the first line.

## SECOND-LINE THERAPY FOR METASTATIC HER2-POSITIVE BREAST CANCER

Therapy of choice in second-line treatment of metastatic HER2-positive breast cancer is trastuzumab emtansine (T-DM1), based on the results of the EMILIA clinical study. In the EMILIA trial, patients receiving T-DM1 lived on average 5.8 months longer (overall survival) compared to patients receiving lapatinib and capecitabine (median OS: 30.9 months versus. 25.1 months). Patients receiving T-DM1 had a 32% lower risk of death compared to patients receiving a combination of lapatinib and capecitabine (HR = 0.68; *p* = 0.0006), and lived significantly longer without their disease worsening (PFS) compared to those receiving lapatinib and capecitabine (PFS 9.6 months vs. 6.4 months) [23].

After no additional safety signals were observed and all side effects were in line with previously reported data from previous studies and given the favorable safety profile appropriate for heavily pre-treated patients, the use of T-DM1 in second-line metastatic HER2-positive breast cancer treatment is highly recommended, both by global breast cancer treatment guidelines (ESMO, ASCO, AGO, NCCN, St. Gallen) as well as panel members participating in the consensus meeting [5,6,24-26] (Table 11).

**TABLE 11:**
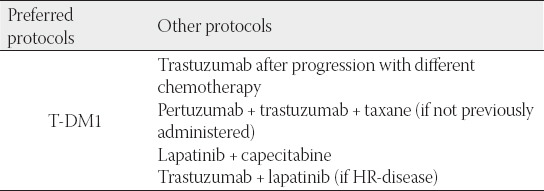
Preferred protocols in second-line treatment of metastatic HER2+ breast cancer

## USE OF BIOLOGICS AND BIOSIMILARS IN THE TREATMENT OF HER2-POSITIVE BREAST CANCER

Treatment of HER2-positive breast cancer today is impossible without targeted anti-HER2 therapy, primarily trastuzumab, pertuzumab, and trastuzumab emtansine. All of these drugs are biologics or monoclonal antibodies, which are produced in living organisms by complex production processes.

A biosimilar is a biologic medical product highly similar to a reference biologic medicine and does not qualify as a generic drug due to differences in raw materials and differences in the manufacturing processes of that biosimilar compared to a ­reference drug of biologic origin [27].

The expert panel agrees that access to biosimilars could potentially reduce the cost of treating HER2-positive breast cancer, but believes it is very important to clearly define how biosimilars can be prescribed and dispensed. Patients deserve the best possible treatment available to achieve better outcomes and potential cure. The introduction of biosimilars can improve the availability of life-saving biological therapies for oncology patients. However, safety considerations and treatment potential should remain a priority and not be outweighed by the potential cost reduction. Accordingly, the expert panel confirmed the position regarding the use of biologics and biosimilars in the treatment of HER2-positive breast cancer and oncology in general:

## POSITION ON THE USE OF BIOLOGICS AND BIOSIMILARS

- As a rule, a biologic that has been proven effective and safe should not be replaced with another biologic.
- A lower price of another drug should not be a reason to change therapy.
- Automatic substitution of biologics (at the pharmacist level) is absolutely unacceptable.
- The treating physician must be able to make a decision to change therapy based on appropriate clinical judgment.
- To ensure drug selection option, it is necessary to insist that the lots for reference drug and for each biosimilar are separated in the tender specifications.
- Patients should have access to the best individual therapy.

## POSITION ON THE USE OF BIOLOGICS AND BIOSIMILARS WITH REGARD TO MARKETING AUTHORIZATIONS

- Biosimilars should be approved in accordance with scientific, rigorous rules for the approval of such drugs, ensuring that there are no significant clinical differences between biosimilars and their reference product in terms of quality, safety, and efficacy. The guidelines prescribed by the WHO and the European Medicines Agency (EMA) should be followed;
- The majority of the expert panel (100% consensus has not been reached) believe that all biosimilars for which a BiH marketing authorization application is submitted should already be approved in the EU through a centralized procedure by EMA, to avoid the possibility of biosimilars of unverified quality/safety/efficacy entering the BiH market, which may endanger the health of patients who need such therapy.
